# Nuclear Magnetic Resonance metabolomics reveals an excretory metabolic signature of renal cell carcinoma

**DOI:** 10.1038/srep37275

**Published:** 2016-11-18

**Authors:** Márcia S. Monteiro, António S. Barros, Joana Pinto, Márcia Carvalho, Ana S. Pires-Luís, Rui Henrique, Carmen Jerónimo, Maria de Lourdes Bastos, Ana M. Gil, Paula Guedes de Pinho

**Affiliations:** 1 UCIBIO@REQUIMTE, Laboratory of Toxicology, Faculty of Pharmacy, University of Porto, Porto, Portugal; 2 CICECO- Instituto de Materiais de Aveiro (CICECO/UA), Departamento de Química, Universidade de Aveiro, 3810-193 Aveiro, Portugal; 3 FP-ENAS, CEBIMED, Fundação Ensino e Cultura Fernando Pessoa, Porto, Portugal; 4 Cancer Biology & Epigenetics Group, Research Center (CI-IPOP) Portuguese Oncology Institute of Porto (IPO of Porto), Porto, Portugal; 5 Department of Pathology, Portuguese Oncology Institute of Porto (IPO of Porto), Porto, Portugal; 6 Department of Pathology and Molecular Immunology-Biomedical Sciences Institute (ICBAS), University of Porto, Porto, Portugal

## Abstract

RCC usually develops and progresses asymptomatically and, when detected, it is frequently at advanced stages and metastatic, entailing a dismal prognosis. Therefore, there is an obvious demand for new strategies enabling an earlier diagnosis. The importance of metabolic rearrangements for carcinogenesis unlocked a new approach for cancer research, catalyzing the increased use of metabolomics. The present study aimed the NMR metabolic profiling of RCC in urine samples from a cohort of RCC patients (*n* = 42) and controls (*n* = 49). The methodology entailed variable selection of the spectra in tandem with multivariate analysis and validation procedures. The retrieval of a disease signature was preceded by a systematic evaluation of the impacts of subject age, gender, BMI, and smoking habits. The impact of confounders on the urine metabolomics profile of this population is residual compared to that of RCC. A 32-metabolite/resonance signature descriptive of RCC was unveiled, successfully distinguishing RCC patients from controls in principal component analysis. This work demonstrates the value of a systematic metabolomics workflow for the identification of robust urinary metabolic biomarkers of RCC. Future studies should entail the validation of the 32-metabolite/resonance signature found for RCC in independent cohorts, as well as biological validation of the putative hypotheses advanced.

Renal cell carcinoma (RCC) is the most common and lethal malignancy of the kidney, accounting for 87% of all renal tumors and representing 2–3% of all human cancers^1^. RCC comprises a range of tumors with distinct histopathological and genetic features, these being traditionally diagnosed by detection of renal masses by ultrasound, computed tomography and magnetic resonance imaging^1^,^2^. However, many renal masses progress while remaining asymptomatic until the late stages of the disease and, consequently, more than 50% of patients are incidentally diagnosed when examined for other clinical purposes and without the suspicion of genitourinary malignancies^2^,^3^. As a result, up to 20–30% of patients present metastatic disease at the time of diagnosis, being less amenable to be cured by surgery and presenting poor prognosis^4^. Therefore, the development of new diagnostic and follow-up methods should certainly have an important impact in RCC clinical management. Possible new strategies may rely on the understanding and measurement of metabolic deviations accompanying the disease, preferably using non-invasive methods. In this context, metabolomics has been increasingly pursued in relation to cancer, either entailing the analysis of tumor tissue (e.g. melanoma^5^, breast^6^,^7^, ovarian^8^, colorectal^9^, brain^10^,^11^,^12^) or of biofluids. The use of biofluid metabolomics in cancer research has consistently increased^13^, minimally invasive samples (plasma/serum and urine) having been used to study ovarian^14^,^15^, colorectal^16^, kidney^17^,^18^,^19^, breast^15^,^20^, lung^21^,^22^ and liver^23^,^24^,^25^ cancers, either using Nuclear Magnetic Resonance (NMR) spectroscopy or Mass Spectrometry (MS). These studies have unveiled potential disease biomarkers but also significant stumbling blocks regarding the impact of systemic effects and confounders e.g. diet, lifestyle, population phenotypes, age, gender^26^,^27^,^28^.

In the particular case of RCC, metabolomics-based studies have already been successfully applied to paired cancer and normal renal tissue^29^,^30^,^31^,^32^,^33^, showing its potential to discriminate cancer and normal tissue as well as study RCC progression and aggressiveness. A lack of correspondence between altered pathways proposed by genomics and metabolomics was observed highlighting that genetics only describes part of the pathophysiologic metabolic rearrangements of RCC^29^. Overall, the studies reported high glutathione levels, high glycolytic and pentose phosphate pathway (PPP) activity as well as alterations in the tricarboxylic acid (TCA) cycle and fatty acid metabolism^29^,^33^. Nevertheless, biofluids such as blood and urine offer the possibility of non-invasive diagnosis and monitoring and these have been the subject of recent metabolomics studies. Serum metabolomics has unveiled changed levels of glucose, glutamine, pyruvate and lactate^34^,^35^ consistent with enhanced glycolytic and glutaminolytic activities, showed potential in nephrectomy follow-up^34^ and patient staging^36^, and more recently produced a possible biomarker cluster of 7 metabolites (alanine, creatine, choline, isoleucine, lactate, leucine, valine) for early RCC^37^. Urine is a particularly suited biofluid concerning kidney cancer, and RCC in particular, due to its intimate contact with the urinary system. A number of MS-based metabolomic studies of RCC urine have been carried out to illustrate the potential of urine metabolic profiling to differentiate RCC patients from controls, two initial reports having considered small sample groups (<10)^17^,^38^ and another MS study addressing the impacts of gender, age and geographical origin on urine metabolic profile and discrimination of cancer from control samples^39^. The latter showed that geographic origin was important whereas the impact of gender and age was residual. Subsequent MS work, using age-, gender- and ethnic-matched cohorts, identified 13 metabolites with statistically different urinary levels between RCC and control samples, and the proposed altered pathways included glycolysis, fatty acid metabolism, amino acid (alanine, aspartate, glutamate) metabolism and nicotinate and nicotinamide metabolism^18^. Further work addressed a xenograft mouse model of highly metastatic RCC and showed that urine is less reflective of tumoral metabolomic changes compared to tissue or serum^19^, nevertheless, increased urinary levels of some acylcarnitines were found in RCC cases, consistent with previous work^40^.

Notwithstanding the important developments described above, NMR-based metabolomics studies of RCC urine has been relatively underexplored^41^,^42^ NMR metabolomics importantly complements MS-based methodologies since, in spite of its lower sensitivity, it enables a wide range of compound families to be detected, with high reproducibility, in a single experiment, thus being particularly suited to untargeted analysis.

This paper reports a NMR profiling study of human urine from a cohort of RCC patients (*n* = 42) and controls (*n* = 49) building on previous work^42^ by adding a systematic evaluation of the impacts of subject age, gender, body mass index (BMI) and smoking habits, to be later considered when defining a RCC metabolic signature. Furthermore, our methodology entailed variable selection of the ^1^H NMR spectra^43^, in tandem with multivariate analysis and validation procedures, to more efficiently retrieve meaningful profiles of metabolite variations related to confounders and disease. Notably, the thus obtained RCC signature was shown to have classification power in unsupervised analysis, for the first time to our knowledge, thus proving its potential in the classification of new subjects prior or irrespective of clinical diagnosis. Finally, a preliminary study of clear cell RCC (ccRCC) compared to other RCC subtypes, and of different RCC stages was performed.

## Results

**Figure 1:**
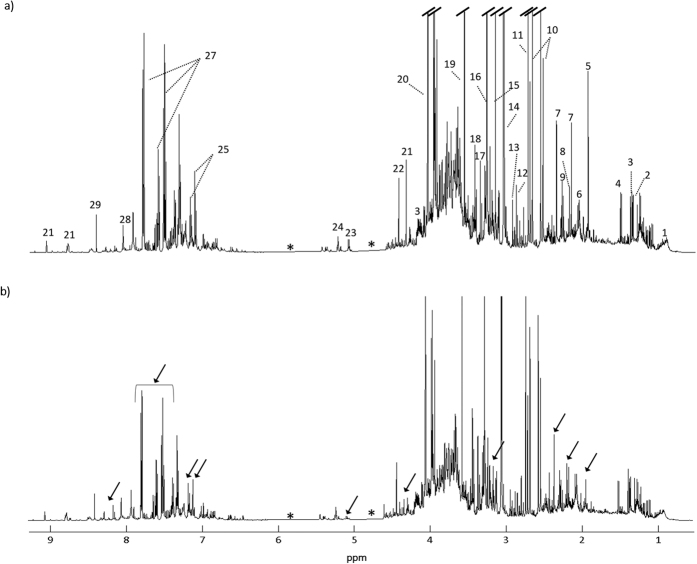
Average ^1^H NMR spectra of urine samples from (**a**) controls and (**b**) RCC patients. Legend: (1) isoleucine, (2) threonine, (3) lactate, (4) alanine, (5) acetate, (6) glutamate, (7) *p*-cresol sulfate (*p*-CS), (8) acetone, (9) valine, (10) citrate, (11) dimethylamine (DMA), (12) trimethylamine (TMA), (13) dimethylglycine (DMG), (14) creatine, (15) trimethylamine-N-oxide (TMAO), (16) 9-methyl-uric acid (tentative assignment), (17) methanol, (18) scyllo inositol, (19) glycine, (20) creatine, (21) trigonelline, (22) trigonellinamide, (23) unassigned (5.11 ppm), (24) glucose, (25) indoxyl sulfate (IS), (26) phenylacetylglutamine (PAG), (27) hippurate, (28) hypoxanthine, (29) formate. Arrows indicate visible spectral alterations. *Excluded regions (water and urea).

Figure 1 shows the average ^1^H NMR spectra of the urine of controls and RCC subjects. A total of 70 metabolites have been identified (Table S1), confirming previous reports of urine NMR assignment^44^,^45^,^46^,^47^. Visual comparison of the spectra in Fig. 1 suggests apparent decreases in acetate, *p*-cresol sulfate (*p-*CS), trimethylamine *N*-oxide (TMAO), hippurate, indoxyl sulfate (IS) and trigonelline, in RCC samples. However, at this stage it is unclear if such changes are statistically meaningful and/or reflect the effect of confounders, in addition to disease. Indeed, differences in subject age (controls: mean age 67, RCC: mean age 60), gender (controls: 34/15 F/M; RCC: 17/22 F/M) or lifestyle factors (e.g. smoking habits, dietary habits) may impact on urine composition. The effect of these may be decreased using matched cohorts, however, this leads to (1) no applicability of models to subjects falling outside the matched range and (2) reduction in sample numbers and, hence, model robustness. In this study, we have used spectral variable selection to reduce the impact of random uncontrolled factors (such as diet and other lifestyle characteristics) and compared the results obtained using an unmatched cohort and an age- and gender-matched sub-cohort (Table 1), while identifying the specific effects of each recognized confounder.

**Figure 2:**
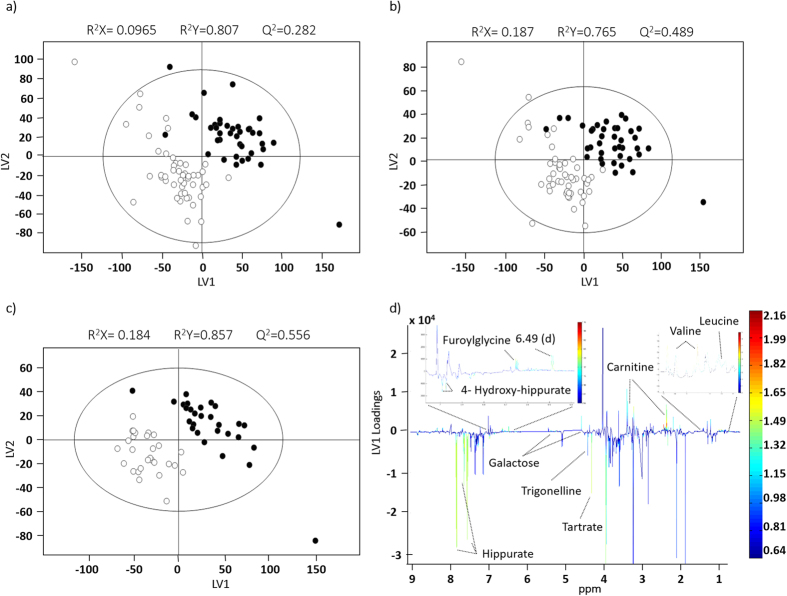
PLS-DA scores scatter plots obtained for the ^1^H NMR spectra of urine of the unmatched cohort: *n* = 49 controls (○) *vs. n* = 39 RCC patients (●), (**a**) without and (**b**) with variable-selection; (**c**) age- and gender-matched sub-cohort: *n* = 28 controls (○) *vs. n* = 28 RCC patients (●), with variable-selection; (**d**) loadings plot corresponding to the model shown in (**b**). All models were obtained with no. LV = 2. The ellipses indicate the 95% confidence limits. The loadings plots are colored according to variable importance to the projection (VIP) and some assignments are indicated.

**Figure 3:**
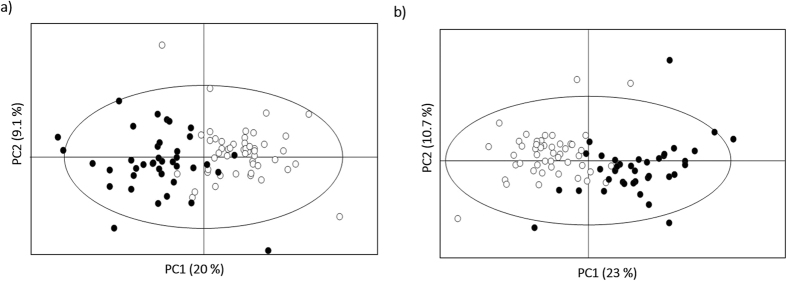
PCA scores scatter plots obtained for the NMR spectra of the urine of the unmatched cohort: *n* = 49 controls (○) *vs. n* = 39 RCC patients (●), (**a**) with the 32-resonance subset and (**b**) with the 23-metabolite signature, i.e. with bias resonances removed. All models were obtained with 2 principal components and the ellipses indicate the 95% confidence limits.

Initial PCA of the full spectra of the unmatched cohort (not shown) did not separate controls from RCC subjects and PLS-DA provided a model with a very low predictive power (median Q^2^ 0.31) (Fig. 2a, Table S2). This result was improved when variable-selected spectra were considered (median Q^2^ 0.59) (Fig. 2b, Table S2), reflecting a reduction of random variability effects, as also expressed by a visible improvement in the Q^2^ distributions plot and ROC curve (Figure S1). When an age- and gender-matched RCC sub-cohort was considered (Table 1), the robustness of the PLS-DA model improved marginally (median Q^2^ 0.67) (Fig. 2c, Table S2), thus indicating that age and/or gender play a minor role in the present context. Nevertheless, the impact of these variables on urine profile was quantified in matched sub-cohorts (Figure S2a,b, Table S2) and a corresponding list of metabolite variations was obtained (Table 2). Generally, age-related changes were indeed small (effect sizes <1), healthy controls over 60 years excreting slightly higher levels of IS, and lower levels of citrate, *cis-*aconitate, creatine, creatinine, hypoxanthine and trigonellinamide and some unassigned resonances. Decreased *cis-*aconitate, creatine and cretinine confirmed previous reports^48^,^49^,^50^,^51^ but the remaining characteristics seem to be specific of the particular cohort considered here. In addition, healthy males were observed to excrete higher amounts of 1-methylhistidine, isoleucine, tartrate and unassigned resonances (Un) 3, 4 and 6 (at δ 0.92, 1.15, 6.19 respectively) and less glutamate, 1,6-anhydroglucose and Un 5 (δ 2.05) (Table 2). Interestingly, these variations are, to our knowledge, novel compared to reported gender-related alterations in urine^48^,^49^,^50^,^51^, thus confirming the need to establish confounders’ impact for each new (unmatched) cohort under study. Furthermore, since the RCC cohort was heterogeneous regarding smoking habits and BMI (Table 1), the impact of these on the metabolic profile of patients was also verified (Table S2). When age- and gender-matched sub-cohorts were used, the robustness of the PLS-DA model related to smoking habits (SH) was not affected whereas the BMI model was improved (median Q^2^ = 0.57; sensitivity = 96%; specificity = 86% and classification rate = 91%). According to the unmatched PLS-DA models, some metabolite variations emerged as possibly related to smoking habits (increased trigonelline, hypoxanthine and a number of unassigned resonances, and decreased 3-hydroxy-butyrate (3-HBA), 4-deoxythreonic acid, allantoin, betaine, guanidinoacetate (GAA) glucose, lactose and *scyllo*-inositol) and to BMI (increased trimethylamine-*N*-oxide (TMAO) and decreased 3-hydroxy-isovalerate (3-HIVA), citrate and succinate (Table 2). Taking the above confounders into account, both unmatched and matched disease cohorts were studied through the corresponding PLS-DA loadings (Fig. 2d, for the unmatched c*o*hort) and spectral integration, while possible bias due to confounders were identified. Overall, variations in 45 metabolites were identified, in addition to changes in several unassigned resonances (Table 3). A total of 32 features exhibited statistical relevance (p < 0.05). These features comprised 20 assigned compounds and were mostly observed in both matched and unmatched cohorts. However, changes in GAA, *scyllo*-inositol and unassigned resonances at δ 4.29, 9.05 were only revealed in the age- and gender-matched cohort, which suggests such changes may become masked in unmatched cohorts. On the other hand, 2-ketoglutarate (2-KG), 4-hydroxyphenylacetate, fumarate, lactate, threonine and trigonellinamide were only observed to change in the unmatched cohort, probably benefiting from the higher sample numbers. PLS-DA model computed using only the set of 32 metabolites/resonances was comparable in performance to that obtained with all selected spectral variables (Table S2), thus electing the 32-resonances subset as a good descriptor of RCC, when compared to controls. This was confirmed by PCA of the 32-metabolite subset (Fig. 3a), which showed very good group separation. Upon removal of bias resonances (those noted with ^c, e, f, g^ in Table 3), the remaining 23 integrals remarkably retained classification power (median Q^2^ 0.75), compared to the 32-resonances model (median Q^2^ 0.68) (Table S2), while PCA improved only very slightly (Fig. 3b), thus again demonstrating that age, gender or smoking habits discrepancies do not hinder RCC classification, based on the urine profiles of the population under study.

Overall, RCC patients were shown to excrete higher levels of 2-KG, *N*-methyl-2-pyridone-5-carboxamide (2-Py), bile acids (tentative assignment), galactose, hypoxanthine (possible confounder), isoleucine (possible confounder), pyruvate, succinate and valine; and lower levels of 4-hydroxyhippurate, 4-hydroxyphenylacetate, acetone, GAA, glycine, hippurate, malonate, phenylacetylglutamine (PAG), tartrate and trigonelline (Table 3). These results partially confirm previous suggestions of a 7- metabolite urinary signature of early RCC^42^, particularly regarding the increases in lactate (not statistically relevant here) and pyruvate (here with *p*-value 5.43 × 10^−7^, compared to 0.010 in ref. 42) and the decrease in hippurate (here with *p*-value 4.60 × 10^−6^, compared to 0.023 in ref. 42). Previously observed variations in creatine, alanine, betaine and citrate were either not confirmed in this cohort or found to have a confounder contribution (namely, decreased citrate was here related to higher BMI, Table 2).

In addition, metabolic differentiation between distinct RCC subtypes and stages was attempted by PLS-DA (Table S2). Unfortunately, the low sample numbers for each of the non-ccRCC types only enabled a preliminary comparison between ccRCC and all other types put together. This revealed small changes (effect sizes <1) in ccRCC cases (higher levels of trimethylamine (TMA), taurine and unassigned doublets at δ 0.75, 0.78 and 1.25, and lower levels of creatine and trigonellinamide), compared to other types (Table 2). The age-matching of ccRCC and other subtypes samples resulted in a slightly improved PLS-DA model (median Q^2^ = 0.78; sensitivity = 95%; specificity = 93% and classification rate = 91% compared to median Q^2^ = 0.63; sensitivity = 92%; specificity = 90% and classification rate = 91% of the unmatched cohort).

## Discussion

**Figure 4:**
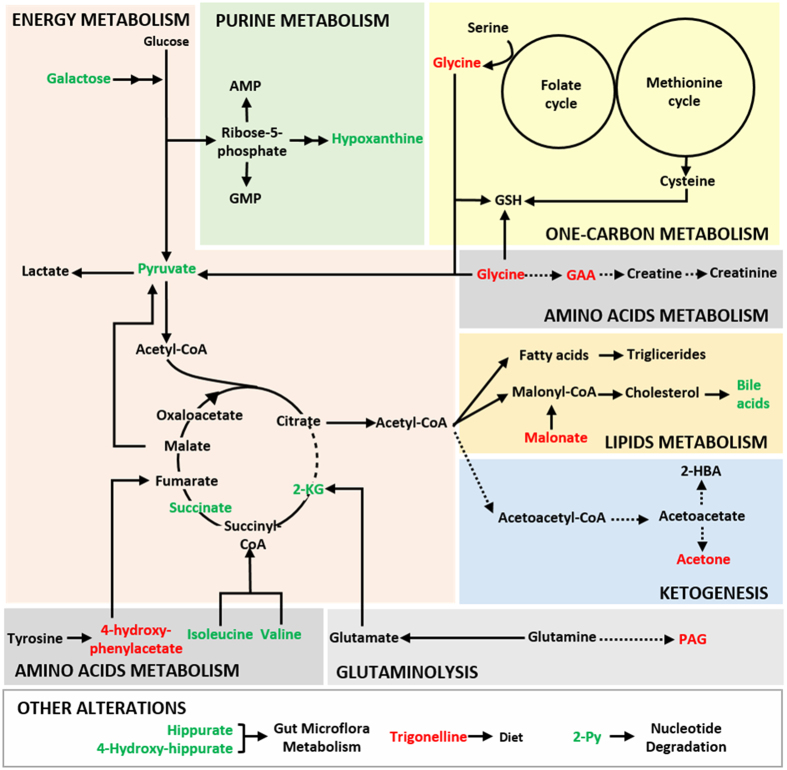
Schematic representation of possibly affected metabolic pathways in RCC. Metabolites found significantly increased and decreased in the urine of RCC patients are presented in green and red, respectively. Metabolic steps hypothesized as up-regulated are presented by bold arrows and those that seem compromised as dashed arrows. 2-HBA: 2-hydroxy-butyrate acid;2-KG: 2-ketoglutarate; 2-Py: N-methyl-2-pyridone-5-carboxamide; AMP:adenosine monophosphate; GAA: guanidinoacetate; GMP: deoxy-guanosine; GSH: gluthatione; PAG:phenylacetylglutamine.

The variable selection strategy used here enabled a 32-resonance urinary signature to be identified as characteristic of RCC urinary profile, compared to controls, and putative biochemical pathways are hereby advanced relating the metabolite changes observed (Fig. 4). Changes in hippurate levels may arise from sources as varied as diet, oxidative stressors and gut microflora and, while hippurate decreases have been reported in a recent account of RCC urine metabolomics^42^ and lung cancer^27^, some inconsistency is found in other cancer studies^52^,^53^,^54^. This indicates the importance of diet/lifestyle parameters in determining the relationship of hippurate and cancer. Furthermore, lower levels of excreted hippurate have also been correlated with type-2 diabetes mellitus^55^, obesity^55^,^56^,^57^ and high blood pressure^58^, the latter two factors being recognized as risk factors of RCC^2^,^4^ and, indeed, characterizing many of the subjects comprised in our RCC group (*n* = 9 obese subjects, *n* = 21 overweight subjects, *n* = 27 subjects with high blood pressure). Trigonelline (or *N-*methylnicotinate) may relate to particular dietary products (e.g. coffee) but may also arise from endogenous niacin methylation. Reduced excretion of this compound was reported in liver cancer patients^59^, ovarian cancer patients^15^, patients with pancreatic ductal adenocarcinoma^60^ and lung cancer^27^. Together with the decreasing tendency of trigonellinamide (or 1-methylnicotinamide), this change suggests some impairment of nicotinate and nicotinamide metabolism^61^. This may impact on the conversion of nicotinamide to 2-Py, catalyzed by poly(ADP-ribose) polymerase (PARP), an enzyme also involved in DNA repair mechanisms, replication, chromatin condensation, cellular response to stress and regulation of apoptosis^62^, thus possibly explaining the increased excretion of 2-Py. Furthermore, altered purine metabolism may lead to elevated hypoxanthine levels, as observed in the present study and previously in the urine of patients with Non-Hodgkin lymphoma^63^. An additional note on the above mentioned reduction in excreted trigonelline relates to this compound being a byproduct of the conversion of S-adenosylmethionine to S-adenosylhomocysteine in the methionine cycle. Indeed, its reduction may also indicate S-adenosylmethionine depletion due to its redirection to help replenish reduced glutathione (GSH) levels^64^, an important cellular antioxidant molecule, believed to be associated with increased production of reactive oxygen species (ROS) in cancer cells. The increased level of galactose may be related to its participation in glycolysis. In fact, increased glycolysis activity is hereby made clear by the higher levels of excreted pyruvate and subsequent altered levels of several tricarboxylic acid (TCA) cycle intermediates, namely 2-KG and succinate. Increased glycolytic flux and altered TCA cycle function are well known hallmarks of cancer, affecting not only cellular energetic efficiency but also anabolic/biosynthetic efficiency^65^,^66^,^67^, since intermediates in these pathways are diverted towards the synthesis of proteins, nucleic acids, lipids, and cholesterol synthesis^66^,^68^,^69^,^70^,^71^,^72^, and generally aid in the maintenance of cellular redox^69^,^71^, genetic and epigenetic status^69^,^73^ required for cancer cells proliferation. In connection to enhanced glycolysis, increased circulating and excreted alanine and lactate levels in RCC have been interpreted as evidence of the expected Warburg effect in cancer cells^37^,^42^, however, in this cohort we have only observed a small increasing tendency for lactate. This suggests that such direct evidence may depend on phenotype and/or become masked by inter-subject variability. In addition, the decreased acetone urinary levels found suggest the inhibition of ketogenesis, consistent with the reported preferential use of acetyl coenzyme A (acetyl-CoA) by cancer cells as precursor of lipids/cholesterol/isoprenoids over the ketogenic pathway^74^. This hypothesis is consistent with the decreased amounts of both acetoacetate and 3-hydroxy-butyrate (3-HBA), also components of ketone bodies, found in cancer patients compared to controls (metabolites not discriminant according to their VIP value). Furthermore, a ketogenic diet (high fat, low carbohydrate content) has shown to have a protective/therapeutic effect on cancer probably because it diminishes the glycolytic flux in cancer cells^75^,^76^.

Metabolic deregulation in RCC seems to impact importantly on the levels of glycine (decreased), isoleucine (increased) and valine (increased). Glycine is an essential amino acid highly consumed by fast proliferating cancer cells^77^,^78^, decreased levels of glycine having also been detected in the urine of prostate cancer patients^79^. As serine, glycine sustains the one-carbon metabolism (folate and methionine cycles) that provides precursors for the biosynthesis of several biomolecules^80^ and the increased uptake of glycine has been suggested to relate to oncogenesis and malignancy in several cancer cell lines^78^. Regarding the branched-chain amino acids valine and isoleucine, their urinary levels have also been found increased in human colorectal cancer^81^ and gastric cancer patients^82^. Both studies have revealed that the levels of these amino acids seem dependent on cancer staging (due to distinct extents of proteolysis^74^) although we have not detected relevant changes in this respect. Other amino acid-related changes comprise those affecting PAG and 4-hydroxyphenylacetate, both decreased. PAG is a downstream metabolite of phenylacetic acid and glutamine and it has been found elevated in the urine of colorectal cancer patients^83^ and decreased in the urine of human bladder cancer^84^ and lung cancer^27^. Lower PAG excretion, as that observed here, may reflect lower glutamine availability due to enhanced glutaminolysis, a well-known metabolic adaptation of cancer cells to sustain TCA cycle activity and amino acids and lipids synthesis^85^,^86^. 4-Hydroxyphenylacetate is a product of tyrosine metabolism and has also been reported as significantly decreased in the urine of breast cancer patients^87^ and elevated in the plasma of dialysis patients^88^. It is possible that the decreased excretion of this compound in cancer patients may relate to kidney impairment as a result of cancer progression. Furthermore, GAA is an intermediate in the biosynthesis of creatine being synthesized mainly in the kidneys and then converted to creatine in the liver^89^. The combination of arginine and glycine to form GAA is considered the rate-limiting step of creatine synthesis, thus, the decreased GAA in urine may be due to an impaired renal function and, its urinary excretion has been found decreased in a variety of renal diseases^90^,^91^,^92^. This is also compatible with the decreased creatinine and creatine excretion found in RCC patients compared to controls, which further suggest an impaired renal function and subsequently clearance of this compounds. Furthermore, GAA was also found decreased in the urine of patients with glioblastoma multiforme^93^ and bladder cancer^94^ compared to controls, which may also be suggestive of altered muscle energy metabolism as a systemic effect of cancer^95^. Finally, the tentative assignment of peaks at δ 0.54 and 0.57 as bile acid resonances suggests an impact of RCC on the endogenous cholesterol metabolism. Bile acids are considered in general as markers of liver injury^96^ and have indeed been found elevated in both serum and urine of patients with hepatocellular carcinoma^97^. However, the particular relationship of these compounds with RCC remains unclear, at this stage.

## Conclusions

The results presented here firstly confirm the importance of evaluating the effects of age and gender (as well as of relevant lifestyle parameters) on urine composition, as part of an initial phenotypic characterization of the specific cohort under study. Based on the evaluation of the impacts of age, gender, smoking habits and BMI on urinary profile, we concluded that the impact of these potential confounders is residual, in this population, in terms of RCC classification, although it determines the exact nature of the retrieved disease signature. Hence, the use of unmatched cohorts was found not to hinder successful RCC classification within the present cohort. The results also show that RCC may be successfully described by changes in a total of 32 compounds/resonances (or 23 resonances, if possible biased variables are excluded). Putative interpretation of the identified metabolites changing in RCC, compared to controls, identified possible unspecific effects involving hippurate, trigonelline and trigonellinamide, thus reflecting the importance of diet and gut microflora, as well as nicotinate and nicotinamide metabolism and anti-oxidative mechanisms as less specific systemic effects of cancer. Additional metabolic effects accompanying RCC, probably of a more specific nature, include disturbances in galactose metabolism, probably in association with the expected enhanced glycolysis activity^34^,^35^. The latter was seen to be accompanied by adaptations of the TCA cycle, ketogenesis, selected amino acid metabolism (glycine, isoleucine and valine), creatine and creatinine metabolism and, possibly, endogenous cholesterol metabolism. These biochemical hypotheses will require future validation through complementary biological measurements, a process which would also benefit from further efforts in the assignment of many important still unassigned NMR resonances. In addition, natural follow-ups of these findings comprise external validation of the 32-resonance RCC signature found here, using external independent sets of subjects of the same geographical origin as the training cohort used here. In fact, the possible dependence of the metabolic signature on geographical origin and, in general, on population phenotype needs to be investigated, as it may explain discrepancies between independent studies and, most importantly, unveil the need to define distinct metabolic biomarkers for different populations.

## Methods

### Subjects

The cohort enrolled in this study comprised thirty-nine patients diagnosed with primary RCC (17 females and 22 males; age range 35–79, average age 60) and forty-nine healthy control (cancer-free) subjects (34 females and 15 males; age range 38–86, average age 67) (Table 1). These groups did not include diabetic patients or subjects suffering from other acute conditions. Table 1 also shows the histopathological types of the RCC tumors diagnosed, TNM staging^2^, and information on subject age, gender, and, in the case of RCC patients, smoking habits and BMI (information not available for control subjects). All subjects signed informed consents and the study was approved by the Ethics Committee of the Portuguese Oncology Institute-Porto (CES76/2012). All the experiments were performed in accordance with the relevant approved guidelines and regulations. The patients provided urine samples preoperatively, none having undergone radiation or chemotherapy treatment. Each subject, either patient or healthy volunteer, provided a sample of first void urine sample (after overnight fasting) in a sterile cup. All samples were then centrifuged (4000 rpm, 20 min, 4 °C) and split into several aliquots transferred into cryovials and stored at −80 °C until NMR analysis.

### Sample preparation

Prior to NMR analysis, urine samples were thawed (room temperature) and centrifuged (8000 rpm, 5 min, 4 °C) to remove cells and other precipitated material. Then, 60 μL of buffer solution (1.5 M phosphate buffer pH 7.0 in D_2_O) containing 0.1% of 3-trimethylsilyl-propionate (TSP), used as chemical shift reference, were added to 540 μL of each urine sample. Fine pH readjustment to 7.00 **±** 0.02 was carried out with 4 M solutions of KOD or DCl. The resultant mixture was centrifuged (8000 rpm, 5 min, 4 °C) and 550 μL were transferred to a 5 mm NMR tube.

### NMR measurements

NMR spectra were acquired at 300 K on a Bruker Avance DRX-500 spectrometer operating at 500.13 MHz for proton and equipped with a 5 mm TXI probe. For each sample, a standard 1D ^1^H NMR spectrum was acquired, using a ‘noesypr1d’ (Bruker library) pulse sequence with water suppression during the relaxation delay (4 s) and mixing time (100 ms). Other acquisition parameters were as follows: spectral width (SW) 10000 Hz, 64 k data points and 128 transients. All free induction decays (FID) were multiplied by a 0.3 Hz line-broadening factor prior to Fourier Transformation (FT). Spectra were manually phased, baseline corrected, and internally referenced to TSP at δ 0.00 ppm (TopSpin 3.2). 2D homonuclear and heteronuclear spectra were recorded for selected samples to aid spectral assignment. Specifically, ^1^H-^1^H NMR total correlation spectroscopy (TOCSY) spectra (‘dipsi2phpr’ pulse sequence) were acquired in phase sensitive mode using time proportional phase incrementation (TPPI) and the MLEV17 pulse sequence for spin locking, with acquisition parameters: 4096 data points in dimension 1 (F1) and 256 data points in dimension 2 (F2), 40 scans and SW 8012.82 Hz in both dimensions, relaxation delay 1.5 s, mixing time of MLEV spin lock 80 ms. ^1^H-^13C^ phase sensitive (echo/antiecho) heteronuclear single quantum correlation (HSQC) experiments were recorded with inverse detection and ^13^C decoupling (‘hsqcetgp’ pulse sequence), a total of 2048 data points in both dimensions, 40 scans and SW 8012.82 Hz (F1) and 20831.98 Hz (F2). A relaxation delay of 1.5 s was employed and a refocusing delay equal to 1/4 ^1^J_c-H_ (1.72 ms) was used. For both TOCSY and HSQC spectra, zero-filling to 1024 data points and forward linear prediction were used in f1 and multiplication by a shifted sinebell-squared apodization function was applied in both dimensions prior to FT and phasing. 1D and 2D spectra were compared to reference spectra in the BBIORFCODE-2-0-0 database (Bruker Biospin, Rheinstetten, Germany), as well as other existing databases^98^ and literature reports^44^,^46^,^47^. Statistical Total Correlation Spectroscopy (STOCSY) was also used to aid peak assignment, based on the fact that the method identifies correlated peak intensities arising from the same molecule, as well as from biochemically related molecules^99^.

### Multivariate Statistical Analysis

The spectral region between 9.40–0.50 ppm was considered for multivariate analysis, after exclusion of residual water (5.48–6.16 ppm) and urea (4.62–5.06 ppm) spectral regions (AMIX software Bruker GmbH). Spectra were aligned using recursive segment-wise peak alignment^100^, normalized by probabilistic quotient normalization (PQN)^101^ (Matlab 7.12.0, The MathWorks, Inc) and scaled to unit variance (UV) (SIMCA-P 11.5, Umetrics, Umea, Sweden). Principal component analysis (PCA) and partial least-squares discriminant analysis (PLS-DA) were applied to the NMR spectra (SIMCA-P 11.5, Umetrics, Umea, Sweden). PLS-DA model robustness was assessed by Monte Carlo Cross Validation (MCCV) using 500 iterations. For each of the 500 randomly generated classification models, Q^2^ values (predictive power), number of Latent Variables (LV), and confusion matrices of original and randomly permuted classes were retrieved. Sensitivity (sens), specificity (spec) and classification rates (CR) were computed and the predictive power of each model was further assessed using a Receiver Operating Characteristic (ROC) map, a function of the true positive rate (TPR or sensitivity) and false positive rate (FPR or 1-specificity). PLS-DA models were considered robust when minimal overlap of the original (alternative hypothesis) and randomly permuted (null hypothesis) Q^2^ distributions were obtained. For variable selection studies, spectral variables were selected through the intersection of three conditions: VIP > 1 and VIP/VIP_cvSE_ > 1 and |b/b_cvSE_| > 1^43^. After variable selection, PLS-DA was reapplied and resubmitted to MCCV. For all models computed, the relevant peaks/metabolites contributing to class discrimination were integrated in the original spectra (AMIX software Bruker GmbH) and PQN normalized (Matlab 7.12.0, The MathWorks, Inc). All integrals were compared through the two-samples Student t-test or the nonparametric analogue Wilcoxon rank sum test (statistical relevance considered for *p* < 0.05, confidence level 95%). Additionally, the Benjamini-Hochberg false discovery rate (BH-FDR) correction method^102^ was used to adjust *p*-values for multiple comparisons (corrected *p*-value = *p-*value*(n/(n-2)), where n = number of metabolites/resonances tested) and a significance cut-off equal to 0.05 considered. Furthermore, effect size values were calculated following the definition given in Berben *et al*.^103^.

## Additional Information

**How to cite this article**: Monteiro, M. S. *et al*. Nuclear Magnetic Resonance metabolomics reveals an excretory metabolic signature of renal cell carcinoma. *Sci. Rep.* **6**, 37275; doi: 10.1038/srep37275 (2016).

**Publisher’s note**: Springer Nature remains neutral with regard to jurisdictional claims in published maps and institutional affiliations.

## Supplementary Material

Supplementary Tables and Figures

## Figures and Tables

**Table 1:** List of urine samples collected for controls and RCC subjects, comprising number of samples, age and gender; and, for RCC patients, histopathological cancer type, TNM staging (information not available for 1 subject) [2], smoking habits (information not available for 1 subject) and body mass index (BMI, in kg.m^−2^, information not available for 3 subjects).

Sample group	no. urine samples^a^	Age range/years	Mean Age ± SD	no. Females	no. Males
Controls (total cohort)	49	38–86	66.63 ± 12.85	34	15
RCC (total cohort)	39	35–79	59.97 ± 11.61	17	22
Clear-cell (ccRCC)	24	41–79	61.88 ± 11.53	6	18
Type 1 papillary (pRCC)	6	53–74	60.00 ± 7.56	4	2
Chromophobe (chRCC)	8	35–71	54.25 ± 14.30	6	2
Unclassified type	1	60	—	1	—
Stage I	21	41–79	58.48 ± 11.40	12	9
Stage II	6	42–78	59.50 ± 11.93	3	3
Stage III	8	35–76	62.88 ± 14.22	2	6
Stage IV	4	50–72	62.75 ± 9.22	0	4
Smokers^b^	12	35–76	57.67 ± 12.40	4	9
Non smokers	26	41–79	61.69 ± 10.96	14	12
BMI ≥ 25	24	35–79	61.33 ± 11.45	11	13
BMI < 25	12	42–77	59.50 ± 11.77	5	7
Matched sub-cohorts					
*Controls vs. RCC, age- and gender-matched*					
Controls	28	38–79	61.68 ± 9.96	16	12
RCC	28	35–79	61.75 ± 10.31	17	12
*Controls* ≤ *60 yr vs. Controls* > *60 yr, matched for male/female proportion*					
Age ≤ 60	20	38–60	53.75 ± 5.33	14	6
Age > 60	29	61–86	75.52 ± 7.96	20	9
*Control Females vs. Control Males, age-matched*					
Female	15	50–86	64.80 ± 12.18	—	—
Male	15	50–85	64.87 ± 12.03	—	—

Information on smoking habits and BMI was only available for RCC patients. SD: standard deviation.

^a^One collection per patient.

^b^Includes smokers (*n* = 5) and former smokers (*n* = 7).

**Table 2:** List of varying metabolites in controls due to different ages (>60 yr and ≤60 yr) and gender (top and middle table sections), and in RCC patients (bottom table section) due to different smoking habits, BMI (≥25 and <25) and histological subtype (ccRCC and other subtypes) along with % variation, effect size (ES), standard errors (SE) and *p*-value.

Metabolite	δ_H_ ppm^a^ (multiplicity)	% variation (±uncertainty)	ES (±ES_SE_)	*p*-value
Metabolite variations in controls > 60 yr (n = 29) *vs.* controls ≤ 60 yr (n = 20)				
Citrate	2.70 (d)	−19.5 (12.3)	−0.51 (0.58)	4.73 × 10^−2^(*)
*cis*-Aconitate^c^	3.12 (d)	−17.2 (5.8)	−1.03 (0.61)	2.44 × 10^−3^
Creatine^c^	3.03 (s)	−5.9 (37.1)	−0.04 (0.57)	3.71 × 10^−2^(*)
Creatinine^c^	4.06 (s)	−21.4 (6.1)	−1.10 (0.61)	2.37 × 10^−4^
Hypoxanthine	8.20 (s), 8.22 (s)	−5.7 (19.9)	−0.07 (0.57)	2.05 × 10^−3^(*)
Indoxyl sulfate	7.70 (d)	17.4 (11.7)	0.43 (0.58)	8.42 × 10^−3^(*)
Trigonellinamide	9.3 (s)	−37.4 (22.2)	−0.64 (0.58)	9.49 × 10^−3^
Un 1	2.07 (s)	−5.5 (3.8)	−0.39 (0.58)	1.38 × 10^−2^(*)
Un 2	7.93 (s)	−1.9 (20.9)	−0.02 (0.57)	2.17 × 10^−3^
Unassigned region	6.40–6.36	−19.7 (10.9)	−0.60 (0.58)	2.05 × 10^−3^(*)
Metabolite variations in control males (n = 15) *vs.* control females (n = 15)				
1-methylhistidine	7.05 (s)	19.3 (8.1)	0.79 (0.74)	4.04 × 10^−2^(*)
1,6-anhydroglucose	5.46 (d)	−20.8 (11.4)	−0.74 (0.74)	3.99 × 10^−2^(*)
4-DTA^b^	1.24 (d)	73.8 (15.3)	1.28 (0.79)	1.66 × 10^−3^
Glutamate	2.04 (m)	−7.6 (3.6)	−0.81 (0.74)	3.51 × 10^−2^(*)
Isoleucine	0.98 (d)	19.5 (6.0)	1.08 (0.77)	4.19 × 10^−3^
Tartrate	4.35 (s)	59.4 (17.1)	0.98 (0.76)	1.86 × 10^−2^(*)
Un 3	0.92 (d)	11.2 (4.5)	0.86 (0.75)	2.71 × 10^−2^(*)
Un 4	1.15 (d)	2.81 (31.4)	0.03 (0.72)	1.40 × 10^−3^
Un 5	2.05 (s)	−10.5 (4.2)	−0.96 (0.76)	1.56 × 10^−2^(*)
Un 6	6.19 (s)	336.5 (133.1)	0.34 (0.72)	2.65 × 10^−2^(*)
Unassigned region	6.43–6.41	−25.4 (9.0)	−1.17 (0.77)	9.68 × 10^−4^
Metabolite variations in smoker + ex-smoker RCC patients (n = 12) *vs.* non-smokers (n = 26)				
3-HBA	1.20 (d)	−18.2 (15.7)	−0.38 (0.69)	2.32 × 10^−2^(*)
4-DTA^b^	1.24 (d)	−25.2 (12.3)	−0.56 (0.70)	4.16 × 10^−2^(*)
Allantoin	5.40 (s)	−25.1 (9.3)	−0.90 (0.71)	6.40 × 10^−3^
Betaíne	3.27 (s)	−37.1 (12.5)	−0.89 (0.71)	1.89 × 10^−3^
GAA	3.81 (s)	−30.5 (10.6)	−0.98 (0.72)	5.45 × 10^−3^
Glucose	5.25 (d)	−46.3 (15.0)	−0.87 (0.71)	1.18 × 10^−5^
Hypoxanthine	8.20 (s), 8.22 (s)	48.3 (28.6)	0.62 (0.70)	4.50 × 10^−2^(*)
Lactose	5.26 (d)	−20.5 (9.3)	−0.62 (0.70)	4.16 × 10^−2^(*)
*Scyllo*-inositol	3.37 (s)	−26.4 (8.4)	−1.06 (0.72)	1.71 × 10^−3^
Trigonelline	9.13 (s)	192.4 (43.2)	1.25 (0.74)	2.16 × 10^−3^
Un 7	1.86 (s)	7.8 (13.0)	0.17 (0.69)	3.87 × 10^−3^
Un 8	1.88 (d)	19.3 (5.7)	1.30 (0.74)	5.10 × 10^−4^
Un 9	2.78 (s)	−22.3 (8.0)	−0.85 (0.71)	2.28 × 10^−2^(*)
Un 10	5.35 (s)	−20.5 (9.3)	−0.81 (0.71)	3.87 × 10^−3^
Un 11	7.94 (s)	52.2 (14.4)	1.21 (0.74)	1.91 × 10^−3^
Un 12	8.66 (d)	57.9 (19.8)	1.09 (0.73)	4.83 × 10^−3^
Un 13	8.79 (d)	239.5 (45.0)	1.22 (0.74)	2.16 × 10^−3^
Un 14	9.05 (s)	99.64 (46.3)	0.59 (0.70)	2.76 × 10^−2^(*)
Unassigned region	8.31–8.24	25.0 (9.0)	0.96 (0.72)	1.79 × 10^−2^(*)
Metabolite variations in RCC patients with BMI ≥ 25 (n = 24) *vs.* BM < 25 (n = 12)				
3-HIVA	2.37 (s)	−10.2 (5.0)	−0.74 (0.71)	3.63 × 10^−2^(*)
Citrate	2.70 (d)	−26.6 (18.3)	−0.55 (0.70)	4.86 × 10^−2^(*)
Succinate	2.42 (s)	−6.2 (5.5)	−0.39 (0.70)	6.20 × 10^−14^
TMAO	3.28 (s)	102.3 (21.2)	0.75 (0.71)	1.49 × 10^−2^(*)
Metabolite variations in RCC patients with ccRCC (n = 24) *vs.* other subtypes (n = 15)				
Creatine	3.03 (s)	−22.8 (47.4)	−0.16 (0.65)	7.58 × 10^−3^(*)
TMA	3.90 (s)	20.7 (7.1)	0.83 (0.67)	1.46 × 10^−2^(*)
Trigonellinamide	9.28 (s)	−31.1 (17.9)	−0.76 (0.67)	4.25 × 10^−2^(*)
Taurine	3.43 (t)	19.0 (8.4)	0.57 (0.66)	4.94 × 10^−2^(*)
Un 15	1.25 (d)	39.6 (9.5)	0.89 (0.67)	1.22 × 10^−3^
Un 16	0.75 (d)	16.9 (20.4)	0.27 (0.65)	1.25 × 10^−2^(*)
Un 17	0.78 (d)	75.6 (17.1)	0.87 (0.67)	1.25 × 10^−2^(*)

^a^Chemical shifts of integrated peaks. s: singlet, d: doublet, m: multiplet. ^b^Tentative assignment.

^c^Variation consistent with previous reports (48–51). 3-HBA: 3-hydroxy-butyrate; HIVA: 3-hydroxy-isovalerate; 4-DTA: 4-deoxythreonic acid: GAA: guanidinoacetate; TMA: trimethylamine; TMAO: trimethylamine-N-oxide. Un i: unassigned spin system i, numbered by order of appearance in table. Only signals with *p*-value < 0.05 are presented.

^(*)^not statistically relevant after BH-FDR correction (45).

**Table 3:** List of varying metabolites in the RCC groups, compared to controls, along with % variation (±% uncertainty), effect size (ES), standard error (SE) and *p*-value.

Metabolite	δ_H ppm_^a^ (multiplicity)	% variation (±ncertainty) (relatively to controls)	ES (±SE)	*p*-value
1-methylhistidine^b^	7.05 (s)	−5.2 (5.9)	−0.20 (0.42)	
2-KG^b^	2.45 (t)	74.3 (6.1)	2.08 (0.52)	1.47 × 10^−12^
2-Py	8.33 (s)	32.3 (12.2)	0.54 (0.43)	8.15 × 10^−3^
3-methylhistidine^b^	8.11 (s)	4.9 (13.3)	0.08 (0.42)	
3-HIBA^b^	1.36 (s)	4.7 (5.6)	0.18 (0.42)	
3-HIVA	2.37 (s)	3.0 (5.1)	0.13 (0.42)	
4-DTA	1.24 (d)	−19.9 (11.0)	−0.41 (0.43)	
4-hydroxyhippurate	7.76 (d)	−51.3 (11.3)	−1.17 (0.45)	2.95 × 10^−9^
4-hydroxyphenylacetate^b^	6.88 (d)	−13.9 (6.6)	−0.47 (0.32)	5.09 × 10^−3^
Acetate	1.93 (s)	−63.2 (37.2)	−0.45 (0.43)	
Acetone	2.24 (s)	−6.6 (7.7)	−0.21 (0.42)	1.54 × 10^−4^
Allantoin^c^	5.40 (s)	6.4 (7.2)	0.19 (0.42)	
Ascorbate	4.53 (d)	−14.4 (7.0)	−0.47 (0.43)	
Bile acid†	0.54 (s)	75.5 (13.7)	0.92 (0.44)	5.51 × 10^−6^
Bile acid†	0.57 (s)	101.0 (15.2)	1.04 (0.45)	1.61 × 10^−7^
DMA	2.73 (s)	−8.1 (8.8)	−0.19 (0.42)	
Citrated,^e^	2.70 (s)	−9.1 (24.0)	0.19 (0.53)	
*cis*-aconitate	3.12 (d)	−5.8 (4.0)	−0.31 (0.42)	
Creatine^d^	3.03 (s)	−4.1 (36.9)	−0.03 (0.52)	
Creatinine^d^	4.06 (s)	−0.5 (6.2)	−0.02 (0.52)	
Formate^d^	8.45 (s)	−9.1 (24.0)	−0.11 (0.52)	
Fumarate^b^	6.53 (s)	16.7 (15.1)	0.24 (0.42)	
Furoylglycine	7.68 (d)	5.8 (3.6)	0.33 (0.42)	
GAA^d^	3.81 (s)	−16.7 (9.2)	−0.53 (0.53)	3.05 × 10^−2(^*)
Galactose	5.28 (d)	55.6 (20.7)	0.50 (0.43)	9.75 × 10^−6^
Glutamate	2.04 (m)	2.8 (2.5)	0.25 (0.42)	
Glutamine	2.44 (m)	8.7 (5.1)	0.36 (0.42)	
Glycine	3.57 (s)	−21.7 (7.1)	−0.71 (0.43)	1.68 × 10^−3^
Hippurate	7.56 (t)	−42.2 (12.9)	−0.87 (0.44)	4.60 × 10^−6^
Hypoxanthine^f^	8.20 (s), 8.22 (s)	23.4 (16.0)	0.29 (0.42)	5.64 × 10^−3^
Indoxyl sulfate	7.70 (d)	4.7 (9.5)	0.11 (0.42)	
Isoleucine^g^	0.98 (d)	13.5 (4.9)	0.56 (0.43)	4.58 × 10^−3^
Lactate^b^	1.34 (d)	1.6 (5.8)	0.06 (0.42)	
Malonate	3.11 (s)	−17.6 (15.0)	−0.27 (0.42)	4.79 × 10^−2(^*)
PAG	7.43 (m)	−11.0 (11.9)	−0.22 (0.42)	2.28 × 10^−2(^*)
Pyruvate	2.41 (s)	32.3 (5.5)	1.15 (0.45)	5.43 × 10^−7^
*Scyllo* inositol^d^	3.37 (s)	−1.3 (6.7)	−0.05 (0.52)	
Succinate	2.42 (s)	16.2 (3.4)	1.00 (0.45)	3.02 × 10^−5^
Tartrate	4.35 (s)	−45.8 (10.8)	−1.10 (0.45)	1.26 × 10^−8^
Taurine	3.43 (t)	−7.5 (7.1)	−0.23 (0.42)	
Threonine^b^	1.33 (d)	−4.0 (9.4)	−0.10 (0.42)	
Trigonellinamide^b^	9.28 (s)	−0.3 (14.0)	−0.01 (0.42)	
Trigonelline^c^	9.13 (s)	−28.8 (16.7)	−0.43 (0.45)	5.22 × 10^−4^
TMA	3.90 (s)	−14.0 (8.2)	−0.36 (0.42)	
Valine	1.05 (d)	14.4 (4.1)	0.73 (0.43)	2.98 × 10^−3^
Metabolite variations in unassigned compounds, ordered by ppm (ppm, multiplicity)				
Un 16	0.75, d	10.6 (13.0)	0.17 (0.42)	
Un 17	0.78, d	−16.0 (15.5)	−0.23 (0.42)	
Un 18	0.83, s	10.2 (7.6)	0.29 (0.42)	
Un 7^c^	1.86, s	−5.0 (7.7)	−0.15 (0.42)	1.27 × 10^−2^
Un 1^c^	1.88, d	−5.8 (3.5)	−0.36 (0.42)	
Un 20	1.96, s	−8.1 (6.6)	−0.25 (0.42)	
Un 5	2.05, s	14.4 (3.4)	0.85 (0.44)	4.77 × 10^−6^
Un 1^f^	2.07, s	11.8 (3.0)	0.80 (0.44)	4.00 × 10^−5^
Un 21^d^	2.17, d	−3.0 (26.9)	−0.03 (0.52)	
Un 22	2.38, s	−18.6 (7.2)	−0.55 (0.43)	1.45 × 10^−3^
Un 23^d^	2.39, s	9.1 (4.8)	0.49 (0.53)	
Un 24	2.50, s	13.5 (3.5)	0.80 (0.44)	3.29 × 10^−4^
Un 25	2.76, s	32.8 (4.1)	1.61 (0.48)	5.99 × 10^−11^
Un 9^c^	2.78, s	11.3 (6.8)	0.34 (0.42)	4.56 × 10^−2(^*)
Un 26^d^	4.29, m	10.3 (4.4)	0.60 (0.54)	3.19 × 10^−2^
Un 10^c^	5.35, s	22.9 (9.7)	0.49 (0.43)	
Un 6^g^	6.19, s	69.5 (13.2)	0.91 (0.44)	1.31 × 10^−6^
Un 27	6.49, d	157.7 (30.6)	0.73 (0.43)	2.52 × 10^−2(^*)
Un 12	8.66, d	6.7 (10.1)	0.15 (0.42)	
Un 28	8.68, s	13.9 (16.6)	0.18 (0.42)	
Un 29	8.70, d	−142.1 (371.2)	−0.25 (0.42)	
Un 13	8.79, d	77.8 (28.5)	0.48 (0.43)	
Un 14d,^c^	9.05, s	−33.8 (29.9)	−0.36 (0.53)	3.67 × 10^−2(^*)
Unassigned spectral regions				
0.87–0.84		−1.7 (5.8)	−0.06 (0.42)	
6.40–6.36^f^		16.1 (12.7)	0.27 (0.42)	
6.43–6.41^g^		−14.1 (9.6)	−0.34 (0.42)	1.25 × 10^−2^
6.46–6.44		26.4 (9.8)	0.53 (0.43)	
8.31–8.24^d^		−1.2 (12.4)	−0.03 (0.52)	

^a^Chemical shifts of integrated peaks; s: singlet, d: doublet, t: triplet; m: multiplet.

^b^Variation only detected in the unmatched cohort but unrelated to age, gender, smoking habits or BMI.

^c^May have contribution from different smoking habits.

^d^Variation only detected in the age- and gender-matched model.

^e^May have contribution from BMI.

^f^May have contribution from higher mean age of controls.

^g^May have contribution from higher proportion of females in controls. 2-KG: 2-ketoglutarate; 2-Py: *N*-methyl-2-pyridone-5-carboxamide; DMA: dimethylamine; PAG: phenylacetylglutamine. Un *i*: unassigned compound *i*, numbering follows that indicated in Table 2. Only *p*-values < 0.05 are indicated.

^(*)^not statistically relevant after BH-FDR correction (45).

^†^Tentative assignment.
