# Supplementary material for: Nuclear Magnetic Resonance metabolomics reveals an excretory metabolic signature of renal cell carcinoma
Source: Sci Rep. 2016 Nov 18;6:37275. doi: 10.1038/srep37275 (PMC5114559; doi:10.1038/srep37275)

Nuclear Magnetic Resonance metabolomics reveals an excretory metabolic signature of renal cell carcinoma

Márcia S. Monteiro[1,*], António S. Barros[2], Joana Pinto[2], Márcia Carvalho[1,3], Ana S. Pires-Luís[4,5], Carmen Jerónimo[4,6], Rui Henrique[4,5,6], Maria de Lourdes Bastos[1], Ana M. Gil[2,*,†], Paula Guedes de Pinho[1,†]

[1]UCIBIO@REQUIMTE, Laboratory of Toxicology, Faculty of Pharmacy, University of Porto, Porto, Portugal.

[2]CICECO- Instituto de Materiais de Aveiro (CICECO/UA), Universidade de Aveiro, Aveiro, Portugal.

[3]UCIBIO@REQUIMTE, Laboratory of Toxicology, Faculty of Pharmacy, University of Porto, Porto, Portugal. FP-ENAS, CEBIMED, Fundação Ensino e Cultura Fernando Pessoa, Porto, Portugal.

[4]Cancer Biology & Epigenetics Group, Research Center (CI-IPOP) Portuguese Oncology Institute-Porto (IPO-Porto), Porto, Portugal.

[5]Department of Pathology, Portuguese Oncology Institute-Porto (IPO-Porto), Porto, Portugal.

[6]Department of Pathology and Molecular Immunology-Biomedical Sciences Institute (ICBAS), University of Porto, Porto, Portugal.

Table S1. List of metabolites assigned in the 500 MHz ^{1}H NMR spectra of human urine from controls and RCC patients, pH 7.00±0.02. s: singlet, d: doublet, t: triplet, q: quartet, dd: doublet of doublets, m: multiplet, br: broad. [a] Metabolites assigned through STOCSY. [b] Tentative assignment. 2-HIBA: 2-hydroxy-isobutyrate; 2-KG: 2-ketoglutarate; 2-Py: *N*-methyl-2-pyridone-5-carboxamide; 3-HBA: 3-hydroxy-butyrate; 3-HIVA: 3-hydroxy-isovalerate; 4-DEA: 4-deoxyerythronic acid; 4-DTA: 4-deoxythreonic acid; DMA: dimethylamine; DMG: dimethylglycine; GAA: guanidinoacetate; IS: indoxyl sulfate; *p*-CS: *p*-cresol sulfate; PAG: phenylacetylglutamine; TMA: trimethylamine; TMAO: trimethylamine-N-oxide.

**Assignment**	**$\delta_{H\ ppm}$ (multiplicity)**
1,6-anhydroglucose	5.46 (s), 3.69 (m), 3.54 (m), 4.10 (dd), 4.62 (dd), 3.76 (dd)
1-methyl-histidine [a]	7.78 (s), 7.05 (s), 3.96 (dd), 3.72 (s), 3.16 (dd), 3.07 (dd)
2-HIBA	1.36 (s)
2-KG	2.45 (t), 3.01 (t)
2-Py	8.33 (s), 7.98 (d), 6.67 (d), 3.63 (s)
3-HBA	1.20 (d), 2.32 (m), 2.42 (m), 4.16 (m)
3-HIVA[a]	1.27 (s), 2.37 (s)
3-methyl-histidine	8.11 (s), 7.15 (s), 3.97 (dd), 3.75 (s), 3.30 (d)
4-deoxyerythronic acid (4-DEA) [b]	1.11 (d), 4.08 (d), 4.11 (m)
4-deoxythreonic acid (4-DTA) [b]	1.24 (d), 3.84 (dd), 4.11 (m)
4-hydroxy-hippurate	7.76 (dd), 6.97 (d), 3.96 (s)
4-hydroxyphenylacetate[a]	6.88 (d), 3.45 (s), 7.16 (d)
4-pyridoxic acid [b]	7.53 (s), 4.51 (s), 2.31 (s)
9-methyl-uric acid [b]	3.16 (s)
Acetate	1.93 (s)
Acetoacetate	2.27 (s)
Acetone	2.24 (s)
Alanine	1.49 (d), 3.78 (q)
Allantoin	5.39 (s)
Ascorbate	4.53 (d), 4.01 (m), 3.77 (m)
Bile acid [b]	0.54 (s)
Bile acid [b]	0.57 (s)
Betaine [a]	3.91 (s), 3.27 (s)
Carnitine [a]	2.43 (dd), 3.23 (s), 3.43 (m), 4.58 (m)
Choline	3.20 (s), 3.52 (m), 4.08 (m)
cis-aconitate	5.72 (t), 3.12 (d)
Citrate	2.54 (d), 2.69 (d)
Creatine	3.03 (s), 3.93 (s)
Creatinine	3.05 (s), 4.06 (s)
DMA	2.73 (s)
DMG	2.93 (s), 3.72 (s)
Formate	8.46 (s)
Fumarate	6.53 (s)
Furoylglycine	7.68 (d), 7.14 (dd), 6.65 (q), 3.94 (d)

Galactose	5.28 (d), 4.60 (d), 4.01 (d), 3.98 (d), 3.92 (d), 3.84 (d), 3.72 (m), 3.64 (dd), 3.49 (dd)
Glucose (α anomer)	4.66 (d), 3.91 (m), 3.73 (dd), 3.50 (t), 3.43 (m), 3.23 (dd)
Glucose (β anomer)	5.25 (d), 3.84 (m), 3.77 (dd), 3.72 (t), 3.54 (dd), 3.42 (t)
Glutamate	3.75 (dd), 2.34 (m), 2.12 (m), 2.04 (m)
Glutamine	3.77 (t), 2.44 (m), 2.14 (m),
GAA	3.81 (s)
Glycine	3.57 (s)
Hippurate	8.53 (s, br), 7.84 (d), 7.64 (t), 7.56 (t), 3.97 (d)
Histidine	7.98 (s), 7.13 (s), 4.01 (dd), 3.29 (s), 3.20 (dd)
Hypoxanthine	8.22 (s), 8.20 (s)
IS	7.70 (d), 7.50 (d), 7.36 (s), 7.28 (dd), 7.21 (dd)
Isoleucine	0.94 (t), 0.98 (d), 1.26 (m), 1.46 (m), 1.99 (m), 3.62 (d)
Lactate	4.11 (q), 1.34 (d)
Lactose	5.26 (d), 4.48 (d), 3.94 (m), 3.86 (m), 3.79 (m), 3.73 (m), 3.66 (m), 3.59 (dd), 3.55 (m), 3.28 (dd)
Leucine	0.95 (d), 1.70 (m), 3.72 (t)
Lysine	1.46 (d), 1.73 (m), 1.93 (m), 3.01 (t), 3.78 (t)
Maleate	6.03 (s)
Malonate	3.11 (s)
Mannose	5.18 (d), 3.92 (m), 3.88 (dd), 3.85 (m), 3.81 (m), 3.74 (m), 3.65 (m), 3.58 (t), 3.38 (dd)
Methanol	3.34 (s)
p-CS	2.35 (s), 7.29 (d), 7.21 (d)
PAG	7.43 (m), 7.37 (m), 4.18 (m), 3.67 (d), 2.28 (t), 2.10 (m), 1.93 (m)
Pyruvate	2.41 (s)
Scyllo-inositol	3.37 (s)
Succinate	2.42 (s)
Sucrose	5.42 (d), 4.24 (d), 4.06 (t), 3.89 (m), 3.85 (m), 3.83 (dd), 3.78 (t), 3.63 (s), 3.56 (dd), 3.48 (t)
Tartrate	4.35 (s)
Taurine [a]	3.43 (t), 3.26 (t)
Threonine	4.26 (m), 3.61 (d), 1.33 (d)
Trigonellinamide	9.28 (s), 8.97 (d), 8.90 (d), 4.50 (s)
Trigonelline	9.13 (s), 8.84 (s, br), 8.09 (t), 4.44 (s)
TMA	3.90 (s)
TMAO	3.28 (s)
Tyrosine	7.20 (d), 6.91 (d), 3.95 (dd), 3.20 (dd), 3.06 (dd)
Urea	5.80 (s, br)
Valine	2.27 (m), 1.05 (d), 0.99 (d), 3.61 (d)
Xylose	5.21 (d), 4.56 (d), 3.93 (dd), 3.63 (m), 3.54 (dd), 3.42 (t), 3.33 (dd), 3.23 (dd)

Table S2. Results obtained by MCCV (500 iterations) of PLS-DA models built for: Controls and RCC patients: unmatched controls *vs* RCC, age- and gender-matched controls *vs* RCC, unmatched controls *vs* RCC with 32 signal integrals with univariate statistical relevance; controls alone: age ≤ 60 yrs *vs* age > 60 yrs, females *vs* males; RCC patients alone: BMI < 25 *vs* BMI ≥ 25, (smokers and ex-smokers) *vs.* non-smokers, non-ccRCC subtypes *vs* ccRCC, RCC stages I and II vs. RCC stages III and IV. Q^2: predictive power, Sens: sensitivity, Spec: specificity, CR: classification rate. * and **: models obtained using the 32 integrals and 23 integrals (no bias resonances), respectively, found to vary with univariate statistical relevance (p-value < 0.05 in Table 3). *** only matching according to age was possible.

	**No variable selection**				**With variable selection**			
	Median Q^2	Sens (%)	Spec (%)	CR (%)	Median Q^2	Sens (%)	Spec (%)	CR (%)
**Control and RCC subjects**								
Controls (n=49) vs RCC (n=39)	0.31	74	91	83	0.59	86	96	92
Controls (n=28) vs RCC (n=28) (age- and gender-matched)	0.21	61	81	71	0.67	85	90	88
Controls (n=49) vs RCC (n=39) using 32 signal integrals *	0.68	86	97	92	-	-	-	-
Controls (n=49) vs RCC (n=39) using 23 signal integrals **	0.75	91	98	95	-	-	-	-
**Control subjects only**								
Age≤60 yrs (n=20) vs age>60 yrs (n=29) (gender-matched)	0.09	65	65	65	0.44	88	94	90
Females (n=15) vs Males (n=15) (age-matched)	-0.29	67	66	66	0.46	85	100	93
**RCC subjects only**								
Non-smokers (n=25) vs smokers (n=12)	0.02	66	90	83	0.41	75	96	89
BMI<25 (n=12) vs. BMI≥25 (n=24)	-0.11	78	27	63	0.45	89	66	81
Other subtypes (n=15) vs. ccRCC (n=24)	-0.04	68	42	58	0.63	92	90	91
Stages I and II (n=27) vs. Stages III and IV (n=12)	-0.22	25	84	66	0.35	55	91	80

Figure S1. List of metabolites assigned in the 500 MHz ^{1}H NMR spectra of human urine from controls and RCC patients, pH 7.00±0.02. s: singlet, d: doublet, t: triplet, q: quartet, dd: doublet of doublets, m: multiplet, br: broad. [a] Metabolites assigned through STOCSY. [b] Tentative assignment. 2-HIBA: 2-hydroxy-isobutyrate; 2-KG: 2-ketoglutarate; 2-Py: *N*-methyl-2-pyridone-5-carboxamide; 3-HBA: 3-hydroxy-butyrate; 3-HIVA: 3-hydroxy-isovalerate; 4-DEA: 4-deoxyerythronic acid; 4-DTA: 4-deoxythreonic acid; DMA: dimethylamine; DMG: dimethylglycine; GAA: guanidinoacetate; IS: indoxyl sulfate; *p*-CS: *p*-cresol sulfate; PAG: phenylacetylglutamine; TMA: trimethylamine; TMAO: trimethylamine-N-oxide.

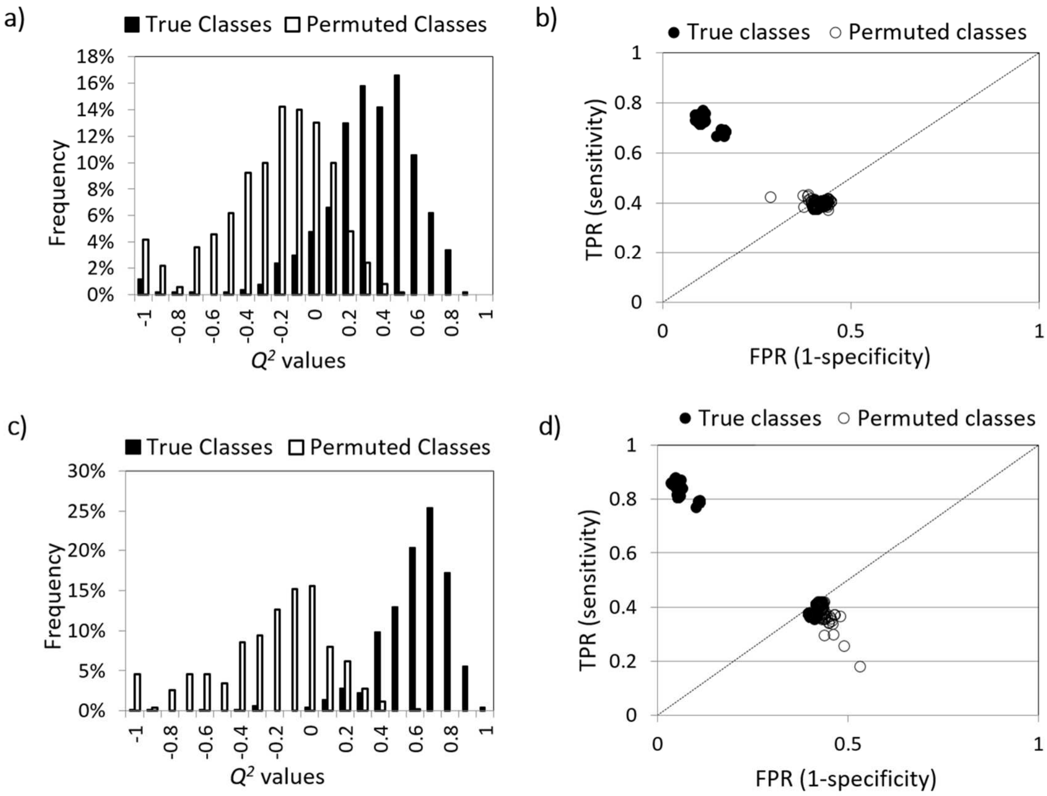

Figure S2. PLS-DA scores scatter plots (left) and corresponding LV1 loadings (right) obtained for variable selected ^{1}H NMR spectra of urine from a) age-matched sub-cohort of control females (o, *n*=15) *vs* control males (●, *n*=15); b) controls with age ≤ 60 (◊, *n*=20) *vs* controls with age >60, (♦, *n*=29). All models were obtained with no. LV=2. The ellipses indicate the 95% confidence limits. The loadings plots are colored according to variable importance to the projection (VIP) and relevant assignments are indicated.

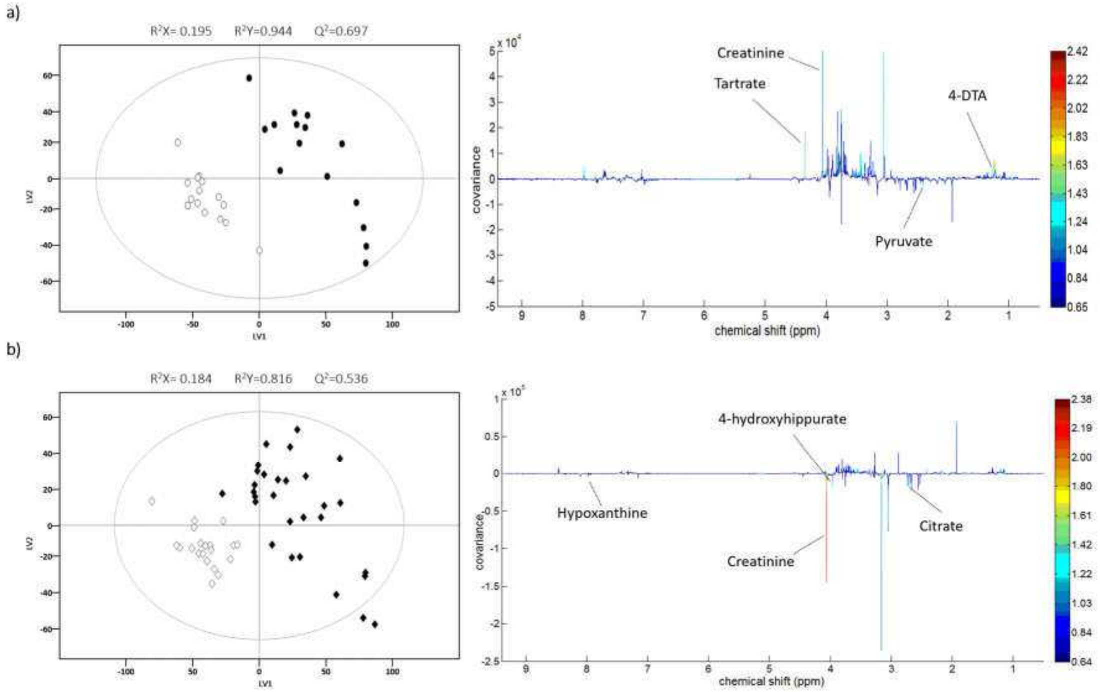

Supplement: Supplementary Tables and Figures [file srep37275-s1.pdf]
